# Inconsistency in English translation of our generalist specialty “Sogo‐Shinryo” among university hospitals in Japan

**DOI:** 10.1002/jgf2.514

**Published:** 2021-12-05

**Authors:** Hideki Tsunoda, Kaku Kuroda

**Affiliations:** ^1^ Department of Family Medicine University of Pittsburgh Medical Center Shadyside Pittsburgh Pennsylvania USA; ^2^ Department of Family Medicine SUNY Upstate Medical University Syracuse New York USA

## CONFLICT OF INTEREST

The authors have stated explicitly that there are no conflicts of interest in connection with this article.

To the Editor,

We read the article by Yuya Yokota with great interest and appreciate the author's effort to describe the role of general medicine physicians in Japan.^1^ I strongly agree that the categorization and lack of consensus of its classification complicate the explanation of “General Medicine.” In addition, we would like to mention the translation issue we face when trying to explain “General Medicine” in English.

The original Japanese word from which the author translated to “General Medicine” is “Sogo‐Shinryo.” As the author mentioned, “Sogo‐Shinryo” is a specialty that includes a variety of generalist physicians such as family physicians, hospitalists, and hospital family physicians. Although the Japanese Medical Specialty Board translated “Sogo‐Shinryo” to “General Medicine” in English,^2^ there is no consensus of the translation so far. We frequently see that each hospital translates “Sogo‐Shinryo” differently depending on the characteristics of the department. This inconsistency generates confusion when we explain the concept of “Sogo‐Shinryo” outside Japan. Hence, we decided to investigate how “Sogo‐Shinryo” is translated in English among university hospitals in Japan.

We searched the official homepage of 82 university hospitals in Japan and examined the English translation of “Sogo‐Shinryo” by the department. If the official homepage of a hospital had no translation, we searched the department homepage that was linked from the official hospital homepage. If the university hospital does not have a “Sogo‐Shinryo” department, but its branch hospital has, we checked the branch hospital's homepage and included the English translation if available. During the process of identification of a “Sogo‐Shinryo” department, we focused on the medical department at university hospitals and did not include divisions of medical universities. We included any department with “Sogo‐Shinryo” in its name, such as “Sogo‐Shinryo‐Naika.” All searches were done on November 16, 2021.

Among 82 university hospitals, 13 were excluded because their “Sogo‐Shinryo” department could not be detected, and 16 were excluded because they had a “Sogo‐Shinryo” department, but no translation was available. Finally, we extracted the translation of “Sogo‐Shinryo” from 53 university hospitals (45 were confirmed on the hospital homepage and eight were confirmed on the department homepage). The results are shown in Table 1. Briefly, most major translations of “Sogo‐Shinryo” were “General Medicine” (*n* = 34, 64% of all). Other translations included “General Medicine and Primary Care” (*n* = 5, 9% of all), “General Internal Medicine” (*n* = 4, 8% of all), and “Family Medicine” (*n* = 2, 4% of all).

**TABLE 1:** Translation of the department of "Sogo‐Shinryo" in English at university hospitals in Japan

The translation of "Sogo‐Shinryo" (*n* = 53)	No.	%
Department of General Medicine	34	64%
Department of General Medicine and Primary Care	5	9%
Department of General Internal Medicine	4	8%
Department of Family Medicine	2	4%
Department of General Medical Department	2	4%
Department of General Clinical Department (Division)	2	4%
Department of Diagnostic and Generalist Medicine	1	2%
Department of Internal and General Medicine	1	2%
Department of General & Family Medicine	1	2%
Department of General Clinics for Outpatients	1	2%

This investigation revealed that our generalist specialty in Japan, “Sogo‐Shinryo,” has a variety of translations in English. This translation inconsistency may reflect the difference in the major elements of work being done by the department and policy of the department.^3^ It is inevitable that there are diverse translations, given the fact that we are now trying to define and describe this category in various way.^1^ We believe that the way of translation will change and become more consistent in future as the role of “Sogo‐Shinryo” is clarified.
